# Sequence Types and Antimicrobial Resistance Profiles of *Salmonella* Typhimurium in the Food Chain in Singapore

**DOI:** 10.3390/microorganisms12091912

**Published:** 2024-09-19

**Authors:** Yen Ching Lim, Kar Hui Ong, Wei Ching Khor, Favian Yue Xuan Chua, Jia Qi Lim, Li Kiang Tan, Swaine L. Chen, Wai Kwan Wong, Matthias Maiwald, Timothy Barkham, Tse Hsien Koh, Joanna Khoo, Joanne Sheot Harn Chan, Kyaw Thu Aung

**Affiliations:** 1 National Centre for Food Science, Singapore Food Agency, 7 International Business Park, Singapore 609919, Singapore; 2 Infectious Diseases Translational Research Programme, Department of Medicine, Division of Infectious Diseases, Yong Loo Lin School of Medicine, National University of Singapore, 1E Kent Ridge Road, NUHS Tower Block, Singapore 119228, Singapore; 3 Laboratory of Bacterial Genomics, Genome Institute of Singapore, 60 Biopolis Street, Singapore 138672, Singapore; 4 Centre for Animal & Veterinary Service, National Parks Board, Singapore 718827, Singapore; 5 Department of Pathology and Laboratory Medicine, KK Women’s and Children’s Hospital, Singapore 229899, Singapore; 6 Department of Microbiology and Immunology, Yong Loo Lin School of Medicine, National University of Singapore, Singapore 117545, Singapore; 7 Duke-NUS Medical School, National University of Singapore, Singapore 169857, Singapore; 8 Department of Laboratory Medicine, Tan Tock Seng Hospital, Singapore 308433, Singapore; 9 Department of Microbiology, Singapore General Hospital, Singapore 169856, Singapore; 10 Department of Food Science & Technology, National University of Singapore, Science Drive 2, Singapore 117542, Singapore; 11 School of Biological Sciences, Nanyang Technological University, 60 Nanyang Dr, Singapore 637551, Singapore

**Keywords:** *Salmonella* Typhimurium, antimicrobial resistance, salmonellosis, surveillance, food, human, One Health

## Abstract

*Salmonella* remains a significant foodborne pathogen globally with *S*. Typhimurium presenting as a frequently occurring serovar. This study aimed to characterize 67 *S*. Typhimurium isolates from humans, food, farms, and slaughterhouses collected in Singapore from 2016 to 2017. Using whole-genome sequencing analysis, the isolates were found to belong to either ST19 (*n* = 33) or ST36 (*n* = 34). ST36 predominated in human intestinal and chicken isolates, while human extra-intestinal and non-chicken food isolates belonged to ST19. Plasmids were predicted in 88.1% (*n* = 59) of the isolates with the most common incompatibility group profiles being IncFIB(S), IncFII(S) and IncQ1. IncFIB(S) (adjusted *p*-value < 0.05) and IncFII(S) (adjusted *p*-value < 0.05) were significantly more prevalent in ST19 isolates, while Col156 (adjusted *p*-value < 0.05) was more significantly found in ST36 isolates. ST36 isolates exhibited higher resistance to multiple antibiotic classes such as penicillins, phenicols, folate pathway inhibitors, aminoglycosides, β-lactam/β-lactamase inhibitor combinations, tetracyclines, and fluoroquinolones. Phylogenetics analysis suggested potential shared routes of transmission among human, chicken, farm and slaughterhouse environments. Taken together, this study offers a cross-sectional epidemiological insight into the genomic epidemiology and antimicrobial landscape of *S*. Typhimurium isolates in Singapore, informing strategies for future public health and food safety surveillance.

## 1. Introduction

*Salmonella* ranks prominently among the frequently occurring foodborne pathogens [1] and is one of the major causes of foodborne diseases in both developing and developed countries [2]. Each year, *Salmonella* is estimated to be associated with 93.8 million incidents of foodborne illness and 155,000 fatalities globally [1,3], highlighting the significance of human salmonellosis as a global public health challenge.

*Salmonella* occurs naturally within environmental ecosystems and is prevalent among both domestic and wild animal populations [3]. *Salmonella* primarily inhabits the intestinal tracts of humans and agricultural animals but can also be found in wild avian species, reptiles, and occasionally, insects [4]. Additionally, feedstuff, soil, and fecal matter have also been frequently recognized as origins of *Salmonella* contamination in agricultural settings [5,6,7]. Being a zoonotic etiological agent, the transmission of *Salmonella* commonly emanates from food products of animal origin [8]. The occurrence of human salmonellosis is frequently associated with the consumption of food contaminated with *Salmonella*. Poultry, pork, and egg products are frequently identified as primary sources of *Salmonella* [9]. The risk for contamination and consequent infections can be further exacerbated by other factors such as ingestion of raw food, poor personal hygiene practices, inappropriate food storage, and improper heat treatment [10,11]. Furthermore, the socioeconomic impact resulting from human salmonellosis extends beyond health-related expenses, which includes additional costs shouldered by business operators for disposing of contaminated food and facing trade limitations [12].

*S*. Typhimurium is one of the most frequently reported *Salmonella* serovars worldwide [13,14]. Similar to other non-typhoidal serovars, the clinical manifestations of *S.* Typhimurium cover a spectrum of symptoms, including nausea, vomiting, diarrhea and abdominal cramps [2]. Although *S.* Typhimurium infections typically cause self-limiting gastroenteritis [15], it can also lead to bacteremia and focal extra-intestinal infections, including conditions such as meningitis and osteomyelitis, particularly among children [16,17]. In addition, the escalating prevalence and spread of antimicrobial resistance within *Salmonella* [10], accompanied by the lack of new antimicrobial agents, presents a global concern. One of the primary causes of antimicrobial resistance is linked to the release of non-metabolized antibiotics or their residues into the environment through feces, along with the misuse or overuse of antibiotics in agricultural or farming practices [18]. Consequently, this engenders genetic selection pressure, fostering the emergence of multidrug-resistant bacterial infections within the community [19]. Resistance to therapeutically relevant antimicrobials will pose significant public health concerns due to its association with higher rates of mortality and morbidity [20,21,22]. Thus, this warrants the need for continuous surveillance for the tracking of trends in the development of antimicrobial resistance and molecular subtypes of the strains to develop tailored treatment and control strategies [17,23].

Several studies, focusing on antimicrobial resistance profiles and molecular subtypes, have been undertaken to characterize *S*. Typhimurium [17,24,25]. Nonetheless, to our knowledge, a study to capture the serovar and antimicrobial profiles of *S*. Typhimurium in Singapore is currently limited. To address the knowledge gap, we performed antimicrobial susceptibility testing and whole genome sequencing on 67 *S*. Typhimurium isolates which are all isolates available from human, food, farm and slaughterhouse environmental samples in 2016 and 2017. Through this study, we identified the prevalent sequence types, plasmid, and antimicrobial resistance patterns of *S*. Typhimurium isolates in Singapore. These findings would be useful to inform strategies for foodborne zoonosis disease surveillance and targeted risk management measures for food safety and public health.

## 2. Materials and Methods

### 2.1. S. Typhimurium Isolates Collection

The study included a total of 67 *S*. Typhimurium isolates, which were available from different sources including farm and slaughterhouse environments (*n* = 10), food (*n* = 38), and humans (*n* = 19) between 2016 and 2017. Human isolates were lab-confirmed cases obtained from KK Women’s and Children’s Hospital, Singapore General Hospital, and Tan Tock Seng Hospital. Food isolates were obtained from routine surveillance and monitoring programs, while farm and slaughterhouse environmental isolates were obtained from the archived bacterial biobanks of the national environmental health laboratories. The collected *S*. Typhimurium isolates were streaked on Tryptic–Soy (Oxoid, Hampshire, UK) or non-selective nutrient agar for further characterization.

### 2.2. Phenotypic Antimicrobial Susceptibility Testing

The minimum inhibitory concentration of various antimicrobials was determined with the MicroScan Neg MIC Panel Type 44 (manufactured by Beckman Coulter, Inc., Brea, CA, USA), which was performed in accordance with the manufacturer’s instructions. The antimicrobial susceptibility profiles of each isolate were interpreted based on the latest versions of the Clinical and Laboratory Standards Institute (CLSI) or EUCAST at the time of analysis. To prevent the overestimation of resistance, isolates with minimum inhibitory concentrations (MICs) falling within the intermediate range were classified as susceptible.

All isolates were subjected to antimicrobial susceptibility testing against 28 antimicrobials belonging to 13 antimicrobial classes: Amikacin; Amoxicillin/K Clavulanate; Ampicillin/Sulbactam; Ampicillin; Aztreonam; Cefepime; Cefotaxime; Cefoxitin; Ceftazidime; Cefuroxime; Chloramphenicol; Ciprofloxacin; Colistin; Doripenem; Ertapenem; Fosfomycin; Gentamicin; Imipenem; Levofloxacin; Meropenem; Minocycline; Nitrofurantoin; Norfloxacin; Piperacillin/Tazobactam; Piperacillin; Tetracycline; Tobramycin; Trimethoprim/Sulfamethoxazole.

### 2.3. Whole-Genome Sequencing (WGS) and Analysis

All 67 *S*. Typhimurium were subjected to whole-genome sequencing (WGS), following previously described protocols [26]. Briefly, 1 mL of each *S*. Typhimurium overnight culture in Universal Pre-Enrichment Broth (Acumedia, San Bernardino, CA, USA) was centrifuged. The bacterial cells were then lysed using enzymatic lysis buffer at 37 °C for 45 min, which was followed by extraction using the DNeasy Blood and Tissue Kit (QIAGEN, Valencia, CA, USA) according to the manufacturer’s instructions. Genomic DNA was sheared using an M220 Focused Ultrasonicator (Covaris, Woburn, MA, USA), and library preparation was carried out using an NEBNext^®^ Ultra™ DNA Library Prep Kit (NEB, Ipswich, MA, USA). Samples were then sequenced on a HiSeq 4000 sequencer (Illumina, San Diego, CA, USA) with 151 bp paired-end reads.

All primary sequence analysis was performed by the Genome Institute of Singapore on the Efficient Rapid Microbial Sequencing Platform (GERMS). The serovar of the isolates was predicted with the SeqSero programme [27], while MLST was called with SRST2 version 0.1.8 [28] by using reference sequences obtained from the PubMLST database for *Salmonella* (http://pubmlst.org/salmonella (accessed on 1 January 2021)). The antimicrobial resistance genes and virulence were also predicted by SRST2 [28], which makes use of the ARGannot resistance gene database (accessed on 1 January 2021)) [29] and the Virulence Factors database (accessed on 1 January 2021) [30] as references, respectively.

Sequencing reads were assembled with Velvet [31] using the VelvetOptimizer helper script (version 2.2.4), which were then passed into the Centre for Genomic Epidemiology (CGE)’s SPIFinder 1.0 (https://cge.food.dtu.dk/services/SPIFinder/ (accessed on 1 January 2021)) and PlasmidFinder 2.1 (https://cge.food.dtu.dk/services/PlasmidFinder/ (accessed on 1 January 2021)) for the identification of *Salmonella* Pathogenicity Islands (SPIs) and plasmids respectively.

Draft de novo genomes were used to build the MLST-specific core genome phylogeny trees using Parsnp version 1.5.3 and HarvestTools version 1.2 with default parameters and auto-assignment of genome as reference sequence [32]. Pairwise SNPs difference between isolates were obtained using mummer [33].

### 2.4. Data Analysis

Statistical significance was calculated using R version 4.2.0. *p*-values below 0.05 were considered significant, and where appropriate, Benjamini–Hochberg correction [34] will be applied. Clustering analysis and heatmaps were generated by the “ComplexHeatmap” version 2.12.1 package [35], while the “Goplot” version 4.2.3 [36] and “ComplexUpset” version 4.2.3 [37] packages in R were used to create the chord diagram for plasmid profiling and Upset plot for SPI profiling, respectively.

Co-occurrence analysis of antimicrobial resistance phenotypes in ST36 isolates was performed by calculating pairwise correlations using the antimicrobial resistance status (presence or absence) of each isolate. The resulting correlation matrix was then visualized with the “Corrplot” version 0.94 package. Correlation analysis was also conducted for antimicrobial resistance genotypes in ST36 by considering the presence or absence of the resistance gene. The correlation was then visualized with the “Ggraph” version 2.2.1 package from R, where only nodes with significant correlation above 0.7 or below −0.7 were displayed.

Non-metric multidimensional scaling analysis (NMDS) for antimicrobial resistance genes was performed using the metaMDS function in the “vegan” version 2.6-8 package using the Jaccard distance metric, wherein the isolates were grouped by their sequence types. Permutational analysis of variance (PERMANOVA) was performed using 10,000 permutations and a Jaccard distance metric.

### 2.5. Ethical Considerations

No ethics approval was required for this study, as the clinical isolates collected from the lab-confirmed cases were anonymized in the study.

## 3. Results

### 3.1. Prevalence of ST19 and ST36 S. Typhimurium Isolates in Singapore

**Figure 1:**
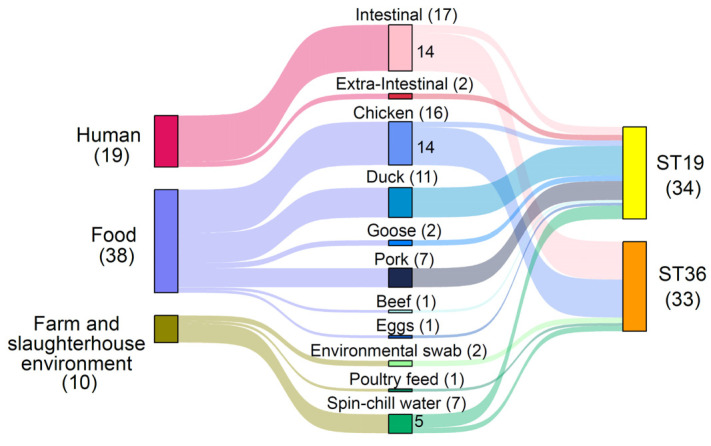
Distribution of *S*. Typhimurium isolates included in the study by their sources and respective sequence types.

All *S*. Typhimurium isolates belonged to two sequence types, ST19 (*n* = 34, 50.75%) and ST36 (*n* = 33, 49.25%) (Figure 1). Most of the human intestinal isolates (14/17, 82.35%) and chicken isolates (14/16, 87.5%) were found to belong to ST36, while all the human extra-intestinal isolates (*n* = 2) and non-chicken food isolates, including duck (*n* = 11), goose (*n* = 2), pork (*n* = 7), beef (*n* = 1) and eggs (*n* = 1), belonged to ST19.

### 3.2. Characterization of Mobile Genetic Factors by Sequence Types

**Figure 2:**
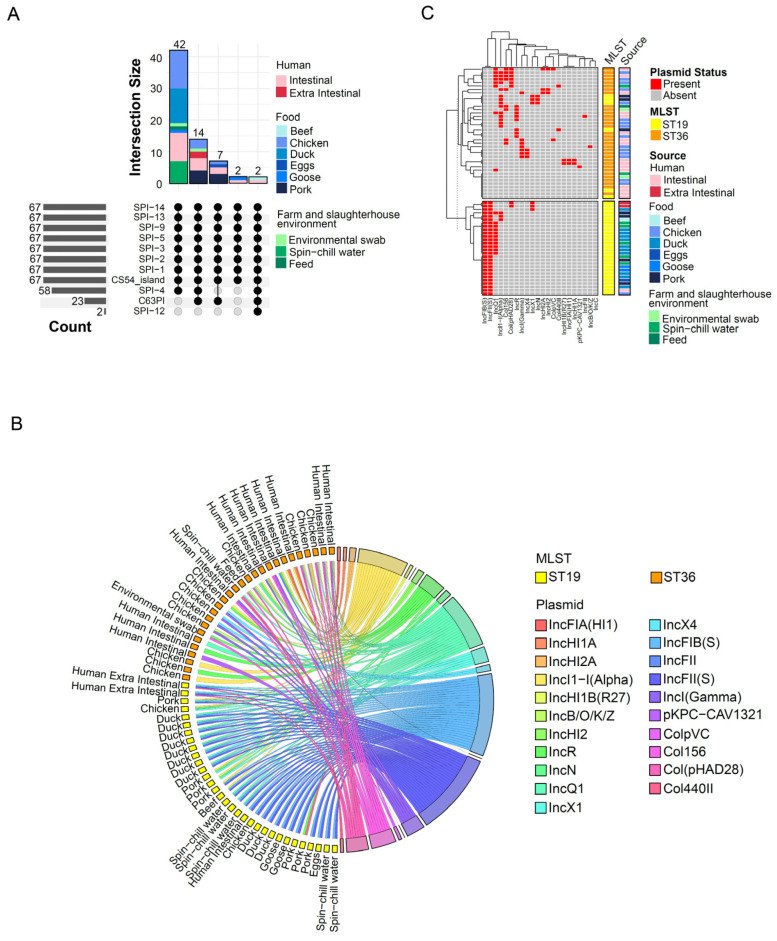
SPI and plasmid profiling. (**A**) UpSet plot of the isolates with detect SPI. The stacked bar (**top**) shows the number of isolates with combinations of detected SPI represented by the matrix (**bottom**). The bar plots on the left indicate the number of isolates with the detection of specific SPI. (**B**) Chord diagram depicting the detection of the specific plasmid (**right**) for each isolate (**left**). (**C**) Plasmids profile of the *S*. Typhimurium isolates.

All the *S*. Typhimurium isolates contained *Salmonella* pathogenicity islands (SPI) SPI-1 to SP-2, SP-3, SP-5, SP-9, SP-13, SP-14, and CS54 (centisome 54 pathogenicity island) (Figure 2A). Only two isolates from human intestinal and beef samples carried SP-12, which were both belonging to ST19. Although C63PI was only found in 34.4% (23/67) of the isolates, this pathogenicity island was found on all human extra-intestinal (*n* = 2) and pork isolates (*n* = 7).

Of the 67 *S*. Typhimurium isolates, 59 were found to harbor at least one plasmid, and the highest detection was found for IncFIB(S) (28/67, 41.8%), IncFII(S) (28/67, 41.8%) and IncQ1 (19/67, 28.4%) (Figure 2B). Comparison of the ST19 and ST36 isolates revealed a significant higher occurrence of IncFIB(S) (Chi-square test, adjusted *p*-value < 0.05) and IncFII(S) (Chi-square test, adjusted *p*-value < 0.05) in ST19 isolates, while Col156 (Chi-square test, adjusted *p*-value < 0.05) was significantly more prevalent in ST36 isolates (Figure 2B and Figure S1). Clustering analysis based on the plasmid profiles exhibited a distinctive sequence type-specific pattern (Figure 2C). The most common plasmid profiles, IncFIB(S)-IncFII(S) (12/67, 17.9%) and IncQ1-IncFIB(S)-IncFII(S) (10/67, 14.9%), were found uniquely in the ST19 isolates (Figure 2C).

### 3.3. Sequence Type Associated Patterns in Phenotypic Antimicrobial Resistance Profile

**Figure 3:**
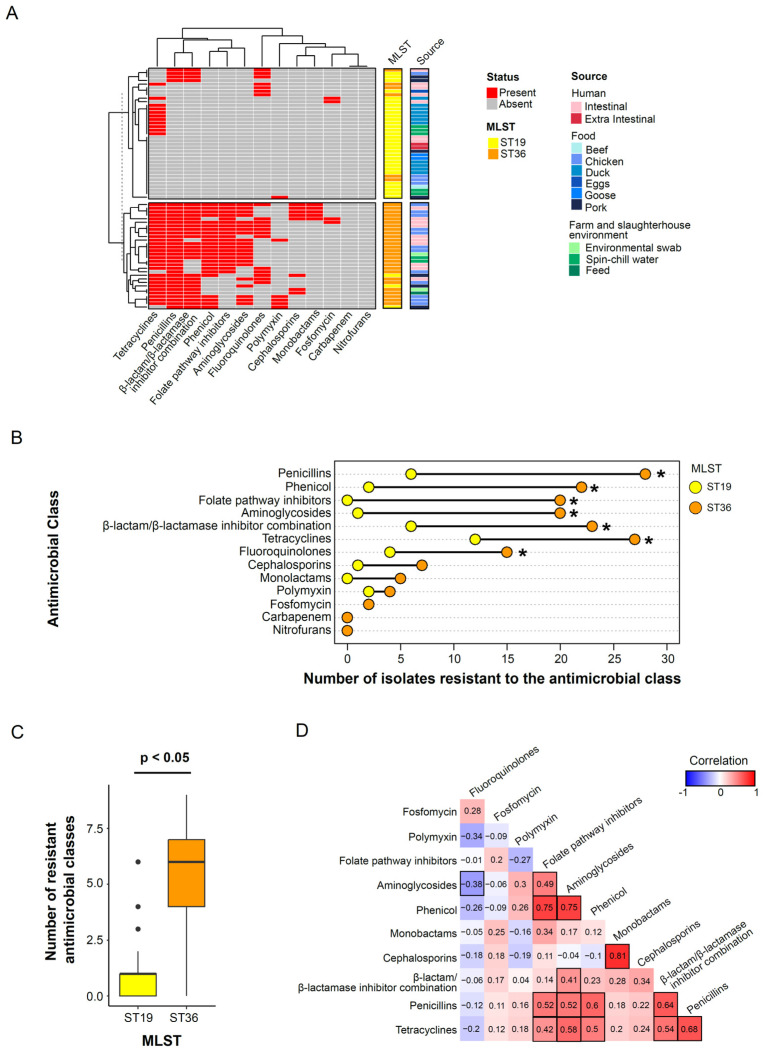
Phenotypic antimicrobial resistance profiling. (**A**) Clustering profile of phenotypic antimicrobial resistance in *S*. Typhimurium isolates. (**B**) Phenotypic antimicrobial resistance in ST19 and ST36 isolates. * Denotes adjusted *p*-value < 0.05, Chi-square test. (**C**) Distribution of the number of resistant antimicrobial classes in ST19 and ST36 isolates. Mann–Whitney U-test *p*-value < 0.05. (**D**) Correlations of phenotypic antimicrobial resistance classes in ST36 isolates. Correlations with *p*-value < 0.05 have been bordered in black.

Phenotypic antimicrobial resistance to at least one tested antimicrobial class was observed in 74.6% (50/67) of the *S*. Typhimurium isolates. All the isolates were susceptible to antimicrobial classes nitrofurans and carbapenem (Figure 3A). Six isolates (6/67, 14.9%), comprising two ST19 pork isolates, three ST36 chicken isolates and one ST36 human intestinal isolate, were phenotypically resistant to polymyxin (colistin) (Figure 3A). Notably, the only two human extraintestinal ST19 isolates included in the study were susceptible to all tested antimicrobial classes. The isolates were most predominantly resistant to β-lactam/β-lactamase inhibitor combination (ampicillin/sulbactam 43.3%), penicillin (ampicillin 50.7%, piperacillin 49.3%), tetracyclines (minocycline 35.8%, tetracycline 53.7%), phenicol (chloramphenicol 35.8%) and aminoglycosides (tobramycin 31.3%, gentamicin 29.9%). It is noteworthy that a substantial proportion of the observed antimicrobial resistance was attributed to ST36 isolates, which exhibited significantly higher resistance than ST19 isolates across a spectrum of antimicrobial classes, including penicillin (Chi-square test, adjusted *p*-value < 0.05,), phenicol (Chi-square test, adjusted *p*-value < 0.05), folate pathway inhibitors (Chi-square test, adjusted *p*-value < 0.05), aminoglycosides (Chi-square test, adjusted *p*-value < 0.05), β-lactam/β-lactamase inhibitor combination (Chi-square test, adjusted *p*-value < 0.05), tetracyclines (Chi-square test, adjusted *p*-value < 0.05) and fluoroquinolones (Chi-square test, adjusted *p*-value < 0.05) (Figure 3B). The number of multidrug-resistant (resistant to ≥three antibiotic classes) ST36 isolates was also significantly higher than those of ST19 isolates (Mann–Whitney U-test, *p*-value < 0.05) (Figure 3C).

To elucidate the correlation patterns of the antimicrobial classes, we constructed a pairwise correlation coefficient matrix for the analyzed antimicrobial classes within the ST36 *S*. Typhimurium isolates (Figure 3D). Corresponding analysis of antimicrobial resistance correlations was not performed for ST19 isolates due to the low antimicrobial resistance observed in this group. A high correlation coefficient exceeding 0.7 was obtained between phenicol and aminoglycosides (R = 0.75, *p*-value < 0.05), phenicol and folate pathway inhibitors (R = 0.75, *p*-value < 0.05), as well as cephalosporins and monobactams (R = 0.81, *p*-value < 0.05), implying a high probability of co-occurrence. Because of the relatively strong correlation between penicillin and tetracyclines (R = 0.68, *p*-value < 0.05), both displayed a similar notable correlation with antimicrobial classes including folate pathway inhibitors, aminoglycosides, phenicol and β-lactam/β-lactamase inhibitor combination. In fact, as many as 39.4% (13/33) of the ST36 isolates were phenotypically resistant in all the six antimicrobial classes (penicillin, tetracyclines, folate pathway inhibitors, aminoglycosides, phenicol and β-lactam/β-lactamase inhibitor combination) (Figure 3A). Among all pairwise comparisons, only aminoglycosides and fluoroquinolones were significantly negatively correlated (R = −0.38, *p*-value = 0.03).

### 3.4. Characterization of Antimicrobial Resistance Genes and Their Concordance with Phenotypic Antimicrobial Resistance Profiles

**Figure 4:**
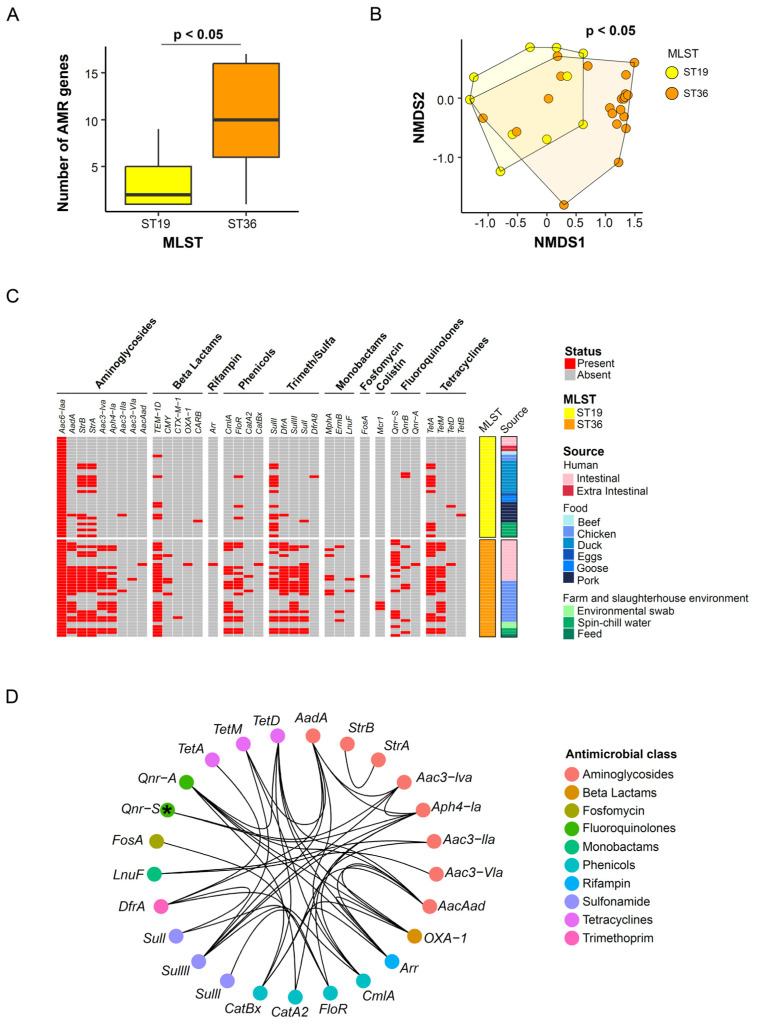
Phenotypic antimicrobial resistance profiling. (**A**) Distribution of the number of detected antimicrobial genes in ST19 and ST36 isolates. * Denotes adjusted *p*-value < 0.05, Mann–Whitney U-test. (**B**) Non-metric multidimensional scaling (NMDS) was performed with the presence and absence status of the antimicrobial genes from all 67 *S*. Typhimurium isolates. Each isolate is represented by a point and colored according to its sequence type. (**C**) Clustering profile of genotypic antimicrobial resistance in *S*. Typhimurium isolates. (**D**) Correlation networks of the antimicrobial genes were constructed using the presence and absence status of the antimicrobial genes from all 67 *S*. Typhimurium isolates. Each node represents a gene and is colored according to its corresponding antimicrobial class. The edges represent the correlation coefficient and are colored based on the strength of the correlation. * Denotes a negative correlation coefficient.

Consistent to phenotypic antimicrobial resistance profile, the number of detected antimicrobial resistance genes was significantly higher in ST36 than ST19 (Mann–Whitney U-test, *p*-value < 0.05) isolates (Figure 4A). Further analysis of the antimicrobial resistance gene profiles revealed significant differences between the ST19 and ST36 isolates (test, *p*-value < 0.05) (Figure 4B).

All 67 analyzed isolates were found to carry at least one antimicrobial resistance gene. The aac6-laa gene was present in all isolates, while other frequently encountered genes included those from aminoglycosides (*aadA*, *strA*, *strB*), β-lactam (*TEM-1D*), trimethoprim/sulfamethoxazole (*sulI*, *sulII*, *sulIII* and *dfrA*). Notably, a colistin resistance gene, mcr1, was identified in three ST36 chicken isolates, which was also phenotypically resistant to colistin. In alignment with phenotypic findings, significantly higher rates of antimicrobial resistance gene detection were found in the ST36 isolates (compared to ST19 isolates), including *aac3-Iva* (Chi-square test, adjusted *p*-value < 0.05), *aph4-Ia* (Chi-square test, adjusted *p*-value < 0.05), *TEM-1D* (Chi-square test, adjusted *p*-value < 0.05, adjusted), cmlA (Chi-square test, adjusted *p*-value < 0.05), *floR* (Chi-square test, adjusted *p*-value = 1.31 × 10^−2^,), *dfrA* (Chi-square test, adjusted *p*-value < 0.05), sulIII (Chi-square test, adjusted *p*-value < 0.05) and *sulI* (Chi-square test, adjusted *p*-value < 0.05). Importantly, the significantly higher levels of antimicrobial resistance gene detection in ST36 isolates were consistent with the antimicrobial susceptibility testing results of the corresponding antimicrobial class obtained previously (Figure 3A).

Given the significantly higher detection of antimicrobial resistance genes in ST36 isolates, we set out to understand the correlation of antimicrobial resistance genes and identify multidrug resistance patterns by constructing a co-occurrence network using ST36 isolates. This network was built based on pairs of antimicrobial resistance genes exhibiting a significant correlation (*p*-value < 0.05) and having correlation coefficients greater than 0.7 or less than −0.7. A total of 37 gene pairs belonging to different antimicrobial classes were identified in our analysis (Figure 4D). Notably, antimicrobial resistance genes *aacAad*, *qnr-A*, *catBx*, *arr*, *sulIII*, *tetD* and *OXA-1* stood out with the highest count of significant correlations within this list. Positive correlations dominate among all gene pairs, except for the *qnr-S* gene, which exhibited negative correlations with *aac3-Iva* (R = −0.7) and *Aph4-Ia* (R = −0.7). This negative correlation was consistent with the findings of the phenotypic co-occurrence antimicrobial resistance analysis (Figure 3C).

### 3.5. Phylogeny of the S. Typhimurium Isolates

**Figure 5:**
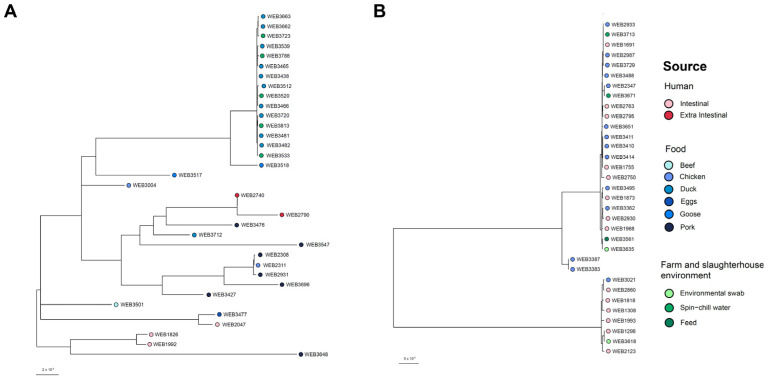
Phylogenetic relationship of *S*. Typhimurium isolates for (**A**) ST19 and (**B**) ST36. Each isolate is represented by a node and colored by the source of origin.

Phylogenetic analysis using core-genome SNPs revealed two well-defined clades, which was distinctively separated isolates of the same sequence type (Figure S2). The ST19 duck isolates formed a cluster with the spin-chill water isolates (0 to 244 SNPs difference), which were in a separate cluster with the human isolates (Figure 5A). In this cluster, 57% (68/120) of the pairwise analysis were within 10 SNPs difference. The ST36 isolates were grouped into two clusters (Figure 5B). One cluster was predominantly composed of human intestinal isolates along with one chicken isolate and one slaughterhouse environment isolate (4 to 351 SNPs difference). The other cluster comprised eight human isolates showing close genetic relatedness with 13 of the chicken isolates and four isolates from the farm and slaughterhouse environment (2 to 495 SNPs difference). Here, only eight out of 224 pairwise analysis were within a 10 SNPs difference with three of them belonging to the human–chicken comparison.

## 4. Discussion

In this study, we characterized 67 locally collected *S*. Typhimurium isolates, which belonged to either ST19 or ST36 (Figure 1). Despite limited sequence type diversity within each food type, the sequence types tended to be associated with food categories. All isolates sourced from ducks, geese, pork, beef, and eggs belonged to ST36, whereas most of the chicken isolates (87.5% (14/16)) were from ST19. Interestingly, most of the human intestinal isolates (14/17) also belonged to ST19, mirroring the predominant sequence type found in chicken isolates. The findings emphasize the need for the enhanced surveillance of chicken meat and their products along the food chain. Furthermore, the results also highlight the importance of implementing appropriate food handling and preparation methods for the prevention of potential *Salmonella* contamination to safeguard public health.

ST19 has been widely recognized as the predominant sequence type within *S*. Typhimurium worldwide [38]. According to the data obtained from the EntroBase database (http://enterobase.warwick.ac.uk, accessed on 13 August 2023), ST19 and ST36 constitute 66.4% and 3.8%, respectively, of the *S*. Typhimurium entries [39]. Although this dataset might not mirror the real-time distribution of sequence types due to its reliance on available SRA data at time of access and the user voluntary upload of data, it provides a useful estimate of the relative prevalence among sequence types. In contrast, our study demonstrates an approximately equal proportion of ST19 and ST36 among *S*. Typhimurium isolates, which deviates from the proportions reported in the EntroBase database. In addition to the limited sample size in our study, which may not be fully representative of the *S*. Typhimurium landscape, the observed phenomenon could be linked to the food consumption profile in Singapore. We observed a high prevalence of ST36 in chicken, which is the most consumed meat in Singapore [11]. In fact, our results showed that most of the human isolates in this study predominantly (14/19) belonged to ST36. The risk of *Salmonella* contamination in food and subsequent infections might also be exacerbated by certain food preparation methods in local cuisine, which tend to involve undercooking chicken for a desired meat texture. Notably, other studies have indicated a convergence or replacement of prevalence between ST19 and other sequence types. For instance, in China, ST34 has gained prominence as one of the most frequent genotypes in clinical samples from patients with diarrhea, making it one of the two sequence types with the highest prevalence alongside ST19 [40]. Similarly, in Mexico, ST213 is replacing ST19 as the most frequently encountered sequence type in both clinical and animal food samples [41]. Changes in the sequence type distribution in different geographical regions may change the local and global epidemiology of sequence type prevalence, and potentially even the emergence of new types that hold considerable epidemiological and public health significance, as they could lead to variations in characteristics, including disease severity, transmission dynamics and treatment strategies.

Adding onto the aforementioned areas of changes in sequence type prevalence trends and the possible emergence of new sequence types with more virulent characteristics, the presence and spread of these multidrug-resistant strains could further limit the available therapeutic options [25]. A study was conducted recently to understand the global burden of antimicrobial resistance by examining 23 pathogens and 88 pathogen–drug combinations across 204 countries and territories. This study estimated that globally in 2019, a median of 1.27 million deaths (with a 95% uncertainty interval of 0.911–1.71 million) was directly attributed to resistance, and 4.95 million deaths (3.62–6.57 million) were associated with bacterial antimicrobial resistance [42]. Additionally, the loss of capital stemming from antimicrobial resistance is projected to range from USD 300 billion to USD 1 trillion worldwide by the year 2050 [43].

Six of the isolates exhibited phenotypic antimicrobial resistance to colistin, including three ST36 chicken isolates harboring the *mcr1* gene (Figure 4C). Additionally, these three isolates also carried the plasmid lncX4 (Figure 2C), which is associated with the dissemination of *mcr1* gene in Enterobacterales [44]. The presence of the *mcr1* gene has also been reported in other *S*. Typhimurium studies involving clinical samples [45], pork offal [46], and an ST19 isolate recovered from a healthy pig in South Korea [47]. As colistin is considered as one of the last-resort drugs, the emergence of resistance to this antibiotic raises concerns for public health.

A high rate of tetracyclines resistance was observed among the ST36 isolates (Figure 3A). Additionally, 35.3% (12/34) of the ST19 isolates carried phenotypic resistance to tetracyclines, which is a rate significantly higher when compared to isolates of the same sequence type. Consistent with our observations, high resistance against tetracyclines has also been reported in other studies [20,26]. These findings warrant attention due to the widespread use of tetracyclines in both human healthcare and animal husbandry. This is attributed to their extensive antibacterial effectiveness, economical production, and absence of severe adverse effects [48,49]. Taken together, this emphasizes the critical importance of close surveillance of the usage and antimicrobial resistance trend for these antibiotics.

Through whole-genome sequencing, we were able to perform SNP-based phylogenetic analysis to achieve higher resolution, providing greater granularity in differentiating isolates of the same sequence type into groups of by their genetic relatedness. The close phylogeny of the clinical, chicken and farm and slaughterhouse environment ST36 isolates (Figure 5B) suggest the possibilities of common chains of transmission and zoonotic transmission. The phylogenetic analysis revealed genetically similar isolates across different sources, suggesting ongoing local transmission events, although the direction of transmission remains to be elucidated. Additionally, the genomic findings presented suggest that chicken meat may play a significant role in the epidemiology of *S*. Typhimurium in Singapore. Taken together, these findings highlight the importance of adopting a One Health approach in developing effective control strategies to reduce *S*. Typhimurium transmission.

Our study is, however, limited by the number of isolates analyzed. A more comprehensive analysis could be achieved by incorporating more isolates over an extended research period. Additionally, including human isolates and data from sporadic cases and outbreaks would contribute to a more robust understanding of the epidemiology of salmonellosis.

## 5. Conclusions

*S*. Typhimurium is one of the most widespread *Salmonella* serovars reported globally. This study characterized 67 isolates from human, food, and farm and slaughterhouse environments in Singapore, identifying two predominant sequence types, ST19 and ST36. The isolates of each sequence type exhibited distinct plasmid and antimicrobial resistance profiles. Furthermore, phylogenetic analysis revealed a close genetic relationship between the isolates from human and chicken and farm and slaughterhouse environments, implying possible transmission routes. These findings underscore the importance of integrated surveillance efforts across One Health sectors for gaining comprehensive epidemiological insights to inform food safety and public health measures.

## Data Availability

The original data presented in the study are openly available in SRA database under Bioproject accession number PRJNA1160349.

## Supplementary Materials

The following supporting information can be downloaded at https://www.mdpi.com/article/10.3390/microorganisms12091912/s1, Figure S1: Proportion of ST19 and ST36 isolates with detection of each plasmid; Figure S2: Phylogenetic relationship of *S*. Typhimurium isolates.
