# Supplementary material for: Sequence Types and Antimicrobial Resistance Profiles of Salmonella Typhimurium in the Food Chain in Singapore
Source: Microorganisms. 2024 Sep 19;12(9):1912. doi: 10.3390/microorganisms12091912 (PMC11434088; doi:10.3390/microorganisms12091912)
Supplement: Supplementary file 1 [file microorganisms-12-01912-s001.zip › microorganisms-3213627-supplementary.pdf]

Supplementary Information

Sequence Types and Antimicrobial Resistance Profiles of Salmonella Typhimurium in the Food Chain in Singapore

Yen Ching Lim[1], Kar Hui Ong[1], Wei Ching Khor[1], Favian Yue Xuan Chua[1], Jia Qi Lim[1], Li Kiang Tan[1], Swaine L. Chen[2,3], Wai Kwan Wong[4], Matthias Maiwald[5,6,7], Timothy Barkham[8], Tse Hsien Koh[7,9], Joanna Khoo[1], Joanne Sheot Harn Chan[1,10], Kyaw Thu Aung[1,10,11*]

[1] National Centre for Food Science, Singapore Food Agency, 7 International Business Park, Singapore 609919, Singapore
[2] Infectious Diseases Translational Research Programme, Department of Medicine, Division of Infectious Diseases, Yong Loo Lin School of Medicine, National University of Singapore, 1E Kent Ridge Road, NUHS Tower Block, Singapore 119228, Singapore
[3] Laboratory of Bacterial Genomics, Genome Institute of Singapore, 60 Biopolis Street, Singapore 138672, Singapore
[4] Centre for Animal & Veterinary Service, National Parks Board, Singapore 718827, Singapore
[5] Department of Pathology and Laboratory Medicine, KK Women's and Children's Hospital, Singapore 229899, Singapore,
[6] Department of Microbiology and Immunology, Yong Loo Lin School of Medicine, National University of Singapore, Singapore 117545, Singapore
[7] Duke-NUS Medical School, National University of Singapore, Singapore 169857, Singapore
[8] Department of Laboratory Medicine, Tan Tock Seng Hospital, Singapore 308433, Singapore
[9] Department of Microbiology, Singapore General Hospital, Singapore 169856, Singapore
[10] Department of Food Science & Technology, National University of Singapore, Science Drive 2, Singapore 117542, Singapore
[11] School of Biological Sciences, Nanyang Technological University, 60 Nanyang Dr, Singapore 637551, Singapore

* Correspondence: aung_kyaw_thu@sfa.gov.sg

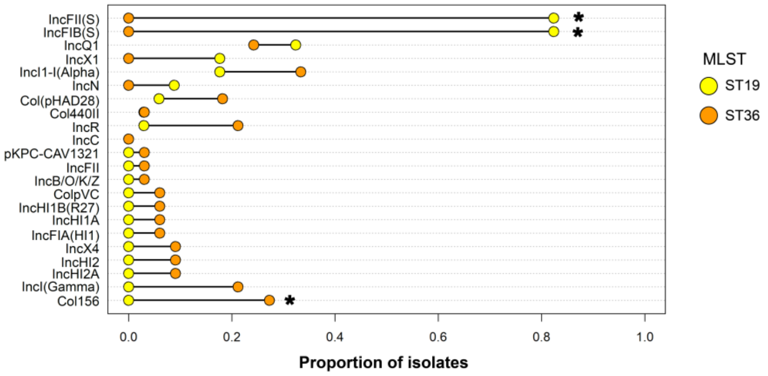

Figure S1: Proportion of ST19 and ST36 isolates with detection of each plasmid. *Denotes adjusted p-value <0.05, Chi-square test.

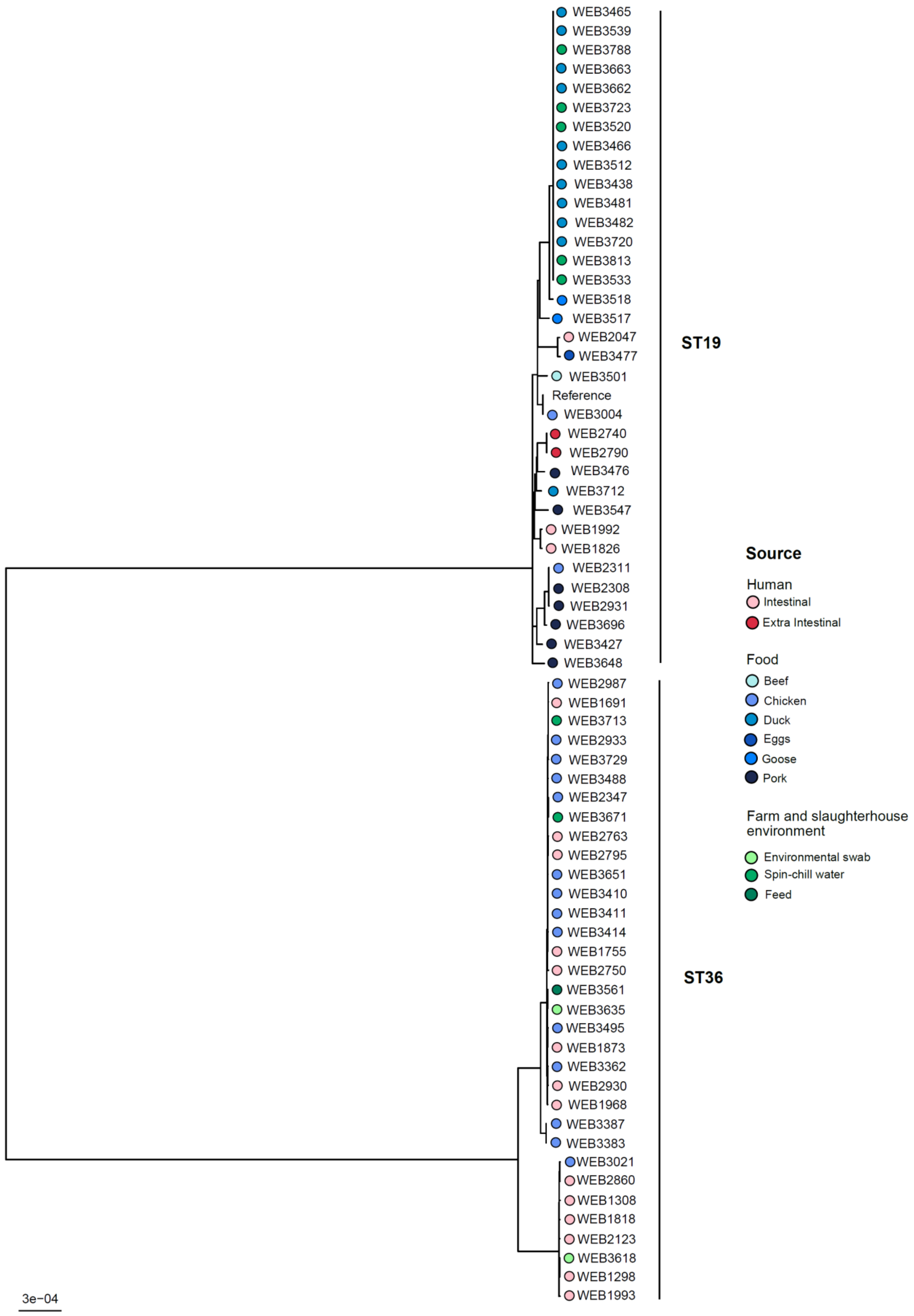

Figure S2: Phylogenetic relationship of *S.* Typhimurium isolates. Each isolate is represented by a node and colored by the source of origin.
